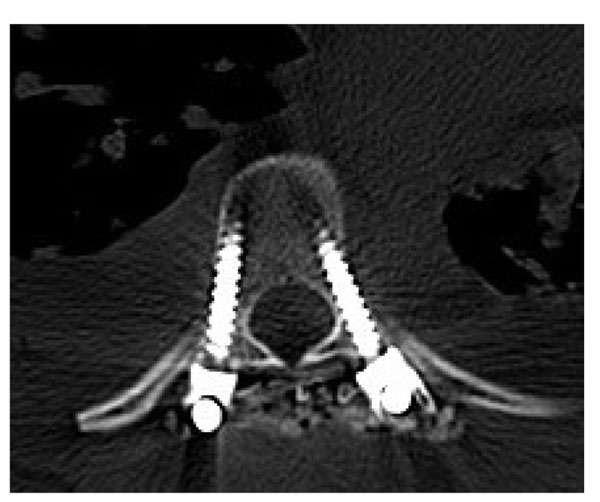# Is there a better derotation manoeuvre in posterior correction of thoracic adolescent idiopathic scoliosis?

**DOI:** 10.1186/1748-7161-10-S1-O69

**Published:** 2015-01-19

**Authors:** Stefano Giacomini, Mario Di Silvestre, Francesco Lolli, Francesco Vommaro, Konstantinos Martikos, Elena Maredi, Andrea Baioni, Tiziana Greggi

**Affiliations:** 1 Deformities of Spine Surgery, Rizzoli Orthopaedic Institute, Bologna, Italy

## Background

Retrospective review of 62 consecutive patients affected by AIS (Lenke type 1 or 2) treated by posterior fusion with pedicle screw-only instrumentation. Three groups identified: Pre-Rod (direct derotation procedure done before inserting rods), Single-Rod (derotation done after concave rod insertion) Double-Rod (after both rods). The Pre-Rods insertion cases showed a significantly better final correction of apical vertebral rotation (61.9% vs 55.8% and 50.1%) and a greater final correction of main thoracic curve. Different manoeuvres can be adopted for direct derotation in posterior correction of thoracic adolescent idiopathic scoliosis (AIS). Aim of the study is to evaluate the better manoeuvre in AIS posterior surgery.

## Materials and methods

62 consecutive patients affected by AIS (Lenke type 1 or 2), were treated by posterior fusion with pedicle screw-only instrumentation, between 2007 and 2009 at one single institution. Three groups were identified: a Pre-Rod group with the direct derotation procedure done before inserting rods (Pre-R group; n=22 patients), a Single-Rod group with the derotation done after concave rod insertion (Single R group; n=20) and a Double-Rod. group after both rods inserted (Double R group; n=20). There were no statistical differences in the 3 groups, in terms of age, Risser’s sign, curve patterns, Cobb main thoracic (MT) curve magnitude and flexibility, extension of fusion, sagittal pre-operative contour and rotation angle (RA sag) of the apical vertebra, measured with axial CT on pre-op and last follow-up control.

## Results

(Average FU 3.6 years, range 2.8 to 4.6). The Pre-Rods insertion cases showed a significantly better final correction of apical vertebral rotation (Pre-R 61.9% Single-R 55.8% Double-R 50.1%; *p*<0.05) and a greater final correction of MT curve (63.4% vs 61.1% and 59.1%; ns) with similar maintenance of initial correction (-1.71° vs -1.78° and -1.73; ns).

The T5-T12 kyphosis angle was similar before surgery (Pre-R 16.9° vs Single-R 17.5° and Double-R 17.2°): it was reduced at final follow-up in Single-R and Double-R cases in comparison with Pre-R patients that presented instead a little increase (19.8° vs 12.5° and 13.5°;ns). Lumbar lordosis was similar before surgery (-42.9° vs -41° and -42.1°) and at final follow-up (-45.1° vs -44.9° and -43.2°; ns). At the latest follow-up, SRS-30 and SF-36 findings were similar between the three groups.

## Conclusions

The direct derotation procedure resulted more effective both concerning correction of apical vertebral rotation and magnitude of MT curve, when applied to the spine before both rods insertion. The hypokyphotic effect of derotation procedure, registered in Single-R and Double-R groups, was avoided doing derotation before rods insertion.

## Consent

Written informed consent was obtained from the patient for the image(s) used in this study. A copy of the written consent is available for review by the Editor of this journal.

**Figure 1:**
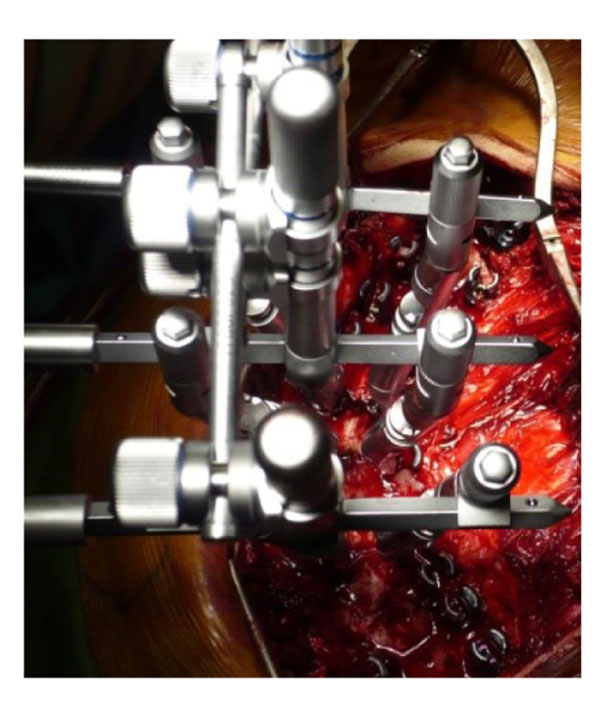


**Figure 2:**